# Engaging Scientists in Science Policy: Experiences From Canada

**DOI:** 10.3389/phrs.2025.1607130

**Published:** 2025-04-25

**Authors:** Steven Lâm, Sarah Raza, Lisa Hansen

**Affiliations:** ^1^ International Livestock Research Institute (ILRI), Nairobi, Kenya; ^2^ Public Health Agency of Canada (PHAC), Ottawa, ON, Canada

**Keywords:** science policy, training, fellowship, environmental scan, public health, scientist, evaluation

## Abstract

**Background:**

Training scientists in science policy is crucial to enhance scientific advice for decision-making. However, there are limited opportunities for scientists to receive such training.

**Analysis:**

We reflected on our participation in a one-year postgraduate science policy fellowship program in Canada. Although recently discontinued in 2023, this fellowship allowed us to refine practical policy skills, contribute to policy outputs that advanced our office’s mandate, and access career pathways beyond academia.

**Policy Options:**

Recognizing the value of engaging scientists in policy, we advocate for continued offerings of science policy training, alongside rigorous evaluation to inform program changes. Additionally, we encourage increased financial support early on for graduate students to sustain a talent pool of scientists who will become future science policy leaders. Lastly, we urge more scientists and students to be active in science policy spaces.

**Conclusion:**

By openly sharing our experiences and learnings from the fellowship, we seek to contribute to ongoing discussions on the importance of science policy training and its role in bridging the gap between science and decision-making.

## Background

While the COVID-19 pandemic increased pressure on governments to transparently develop and communicate science-informed guidance [1, 2], global evaluations of government science advisory mechanisms during this crisis indicated performance fell short of expectations [3]. Policymakers faced criticisms for assuming scientific evidence would enable timely policy solutions and for over-reliance on advice from a select group of scientists [4]. These critiques underscore the need for enhanced science policy relationships, leading to more effective development and delivery of policies.

Strengthening the knowledge base on science policy is important for supporting advisory mechanisms. Science policy, as an area of public policy, is a system whereby the two processes are under the influence of each other [5]. Broadly categorized, it involves (i) *policy for science*, encompassing aspects like research funding and use, and (ii) *science for policy*, elucidating the role of contributions from scientific experts and evidence to policymaking processes. When the goals of scientists and policymakers align, the implementation of scientific advice and policies becomes more effective.

Well before COVID-19, there was a recognized need for adopting interdisciplinary and transdisciplinary approaches to address complex science policy issues [6]. Demand persists for researchers with diverse technical and methodological expertise, along with the ability to collaborate across disciplinary silos, to drive progress in this field. Importantly, there is a need for ‘transition’ programs for new science PhDs, helping scientists develop policy skills not commonly taught in science PhD programs, and connecting policymakers with scientific expertise.

There are few opportunities for science policy training for scientists [7]. While members of academic institutions with links to the policy world could facilitate opportunities for students, structured initiatives are key for expanding the calibre of trainees. However, these initiatives often have limited spots [8]. Additionally, while there are many degree programs related to science policy, it remains true that many science PhDs are directed toward academic research careers. Given that many trained scientists ultimately do not pursue research careers, providing science policy experiences create avenues for them to contribute their skills to policy advisory systems.

This commentary contributes the experiences of two early-career scientists (SL, SR) and a government scientist (LH) who participated in the Mitacs Canadian Science Policy Fellowship program, designed to enhance capacity in support of evidence-informed policymaking in Canada, in the September 2022 to August 2023 cohort. The cancellation of the subsequent program cycle prompted us to reflect on the challenges and opportunities of science policy training. While we draw from our experiences working in a national public health institute in Canada, our reflections are relevant to various contexts. We hope this piece stimulates dialogue on training to support emerging voices of science, and through this, more robust science policy.

### Building Science Policy Capacity in Canada

Canada, like many high-income countries, faces challenges arising from the expansion of its research ecosystem, marked by a growing number of graduate students and postdoctoral fellows [9]. However, the availability of graduate-level positions has not kept pace with this surge, and the uncertain job market is unlikely to accommodate the increasing supply of new graduates in many fields. Additionally, despite undergoing extensive training and producing high-impact research, early-career researchers may not perceive academia as a viable or attractive career path [10, 11].

Facing an uncertain academic job market, and considering alternative opportunities, early-career scientists equipped with graduate training are seeking to acquire new skills and pursue employment in fields where they can apply their expertise to “real world” problems. This includes scientists who are interested in navigating the science policy landscape and learning how to effectively integrate science into decision-making processes [12]. Concurrently, governments are interested in recruiting talented researchers, considering skilled labour gaps and succession planning for scientific leadership roles [13].

While existing mechanisms, including public service recruitment programs, contribute to aligning supply and demand, purposeful training can more thoroughly prepare individuals for these roles. However, most are one-off short trainings and few offer hands-on activities [14]. In Canada, the only long-term, experiential program available to scientists was the Canadian Science Policy Fellowship. It was managed by Mitacs, a national not-for-profit organization that has delivered research and training programs in Canada since 1999. Launched in 2016, the Canadian Science Policy Fellowship aimed to address critical societal challenges by bringing together experts with policymakers.

We were a part of the 6th cohort of the fellowship program. In September 2022, 20 PhD-holders from diverse disciplines were competitively selected to participate in a 12-month fellowship with municipal, provincial, and federal government departments across Canada [15]. Fellows were offered various supports, including workshops on public policy and professional development, as well as sponsorship to attend a national science policy conference. Through hands-on training, the fellowship strived to improve the transfer of specialized knowledge between researchers and policymakers, supported by financial and technical resources.

While such fellowship programs provide valuable experiential learning, it is important to note that they are limited in scope and size and cannot address the full array of organizational needs and professional development demands. There exists a need for a larger population of scientists to access training beyond the scope of fellowships–such as university programs and continuing professional development. Furthermore, with the cancellation of the fellowship program in the 2023 program cycle, there are currently no long-term science policy training options in Canada.

Aside from this fellowship program in Canada, there are at least 10 science policy fellowship opportunities available globally. We conducted a scan of the online grey literature to compile information on these opportunities, presenting a summary in Table 1 [16]. Our internet search employed a combination of keywords, including science policy, training, and fellowship. We excluded programs specific to a single institution, targeted mid/senior-level professionals, or had durations shorter than 3 months. We captured details of the training opportunities, such as country, objective, and funding.

**TABLE 1:** Overview of science policy training programs (global, 2025).

Fellowship name	Country/Region	Discipline	Objective	Duration	Eligibility	Funding for participants
Science and Technology Policy Fellowship	United States	All disciplines	To learn first-hand about policymaking and contribute their knowledge and analytical skills to the policy realm	12 months	Recent doctoral graduates to faculty and retired scientists	US 89–116,000; funded by American Academy for the Advancement of Science
Christine Mirzayan Science and Technology Policy Graduate Fellowship	United States	All disciplines	To obtain the essential skills and knowledge needed to work in science policy at the federal, state, or local levels	3 months	Graduate students, postdoctoral students, professional students, or recent graduates	US 11,000 stipend; funded by National Academies of Sciences, Engineering, and Medicine
Science Policy Fellowship Program	United States	Biomedicine	To understand issues in biomedical research policy by working with the leadership and staff of the Federation and its member societies	3 months	Graduate students and postdocs	None; supported by Federation of American Societies for Experimental Biology
Sea Grant Knauss Marine Policy Fellowship	United States	Marine sciences	To offer experience to graduate students working in ocean, coastal, and Great Lakes management and research	12 months	Graduate students with an interest in ocean, coastal, and Great Lakes	US 73,000; funded by National Oceanic and Atmospheric Association
Genetics and Public Policy Fellowship	United States	Genetics and genomics	To give genetics/genomics professionals an opportunity to contribute to the policymaking process	24 months	Advanced degree holders with an interest in human genetics or genomics	US 79,000 per year; co-funded by American Society of Human Genetics and National Human Genomic Research Institute
Science Policy Fellows Program	United States	Entomology	To teach entomologists the skills needed to successfully advocate for the discipline	24 months	Graduate students, postdocs, mid-career scientists, senior scientists	All travel expenses for workshops and meetings are covered; supported by Entomological Society of America
Congressional Science Fellow	United States	Geological sciences primarily	To allow the federal government to more effectively use scientific knowledge and provide scientists with the opportunity to contribute to public policy	12 months	Primarily geoscientists with a doctorate	US 68,000; funded by American Geophysical Union
Canadian Science Policy Fellowship	Canada	All disciplines	To help solve policy challenges in Canada	12 months	PhD holders from all disciplines	CAD 70–80,000; funded by host organizations
Australian Science Policy Fellowship Program	Australia	All disciplines	To provide a pathway for early-to-mid career scientists to work in federal policy	12 months	PhD holders from all disciplines	AUD 89,000; funded by the Australian government
Policy Fellowships	United Kingdom	All disciplines	To address pressing national and global challenges	18 months	Hold a PhD or equivalent experience	£ 170–210,000 to the host organization; funded by UK Research and Innovation
Science, Technology, and Policy Fellowship	Latin America and the Caribbean	All disciplines	To inform decision-making on global environmental change and sustainable development issues of national priority	12–24 months	Final year of PhD or within 7 years of graduation	US 24,000 per year; funded by Inter-American Institute for Global Change Research

Most programs are offered in the United States. Programs such as the Science and Technology Policy Fellowship offer year-long experiences for doctoral graduates and professionals to develop policy skills. Specialized fellowships exist for fields like biomedicine, marine sciences, genetics, entomology, and geological sciences. Outside the United States, similar opportunities exist, including the Science Policy Fellowship in Australia, and the Research and Innovation Policy Fellowships in the United Kingdom, and the Science, Technology, and Policy Fellowship in Latin America and the Caribbean.

## Analysis

### Experiences of Participating in a Science Policy Fellowship

As Canadian Science Policy Fellows, the authors (SL and SR) were recruited into positions in the Office of the Chief Science Officer in the Public Health Agency of Canada (PHAC). The fellowship offered us opportunities to learn about how science policies are developed and implemented in a science-based governmental agency, as we applied our critical appraisal and synthesis skills to immediate needs, conducting rapid scans of emerging scientific evidence, and connecting research findings to policy implications.

We developed a more concrete understanding of how science support systems and science communications function in a federal agency. Resonating with the experiences of fellows in the United States [17, 18], we found this engagement provided practical policy and communication skills. We were able to pursue additional internal training opportunities and contribute to various outputs, including policy briefs and informational slide decks, advancing our office’s mandate.

Another substantial benefit provided by the fellowship was expanding our professional networks. Our cohort is diverse, emphasizing the program’s commitment to equity. Following interactions with other fellows at the national science policy conference, we established a group chat for sharing updates and opportunities. These connections have enabled us to leverage the experiences of our peers, both among those who have chosen to continue their work within government and those who have opted to pursue academic careers.

While fellows had the opportunity to contribute to various portfolios, gaining insights into departmental priorities and operations, there was an emphasis on cultivating communication and other generalist skills essential for a policymaking career [19]. Through this experience, we learned a distinct way of communicating–one not typically emphasized in academic training–being concise, nimble, and clearly defining next steps.

We also appreciated the chance to collaborate directly with the Chief Science Officer of PHAC on initiatives to advance science. Expanding on this, engagement with policymakers or have opportunities for shadowing would have been valuable. While most scientists recognize the complexity of policymaking, firsthand experience in these spaces can provide deeper insight and help initiate relationship-building between scientists and policymakers [20].

Furthermore, greater flexibility to engage in projects aligning with our technical or methodological expertise could have been helpful. Consideration of specialist skills at the organizational level could involve allocating a certain percentage of time to a research project. Alternatively, fellows could be encouraged to collaborate with full-time scientists, helping to ensure not only science continuity but also the integration of policy within it.

Of note, given the short 12-month duration of the fellowship, a strong onboarding program was essential to help fellows navigate administrative tasks and gain a clear understanding of the host organization’s mandate, structure, and science policy priorities. While supervisors were encouraged to explore fellows’ learning goals, the host organizations must provide projects where fellows can apply their learning and produce tangible results, benefiting both parties. Recruiting scientific specialists for this relatively short time helped to establish a culture of scientific rigour in policy matters as well as enhance the office’s credibility when communicating policy decisions.

## Policy Options

Like scientific research, policy work demands skills in project management, synthesizing complex information, and collaboration. We appreciated the value of science policy training in providing opportunities to use research skills in a new context. We encourage universities, governments, and non-governmental organizations to prioritize access to science policy training, which will not only empower graduates to translate scientific knowledge into policies but will also enhance their employability [11]. Importantly, maintaining a focus on accessibility is crucial to ensure that a diverse group of graduates can actively participate in these training initiatives.

Support for policy fellows with advanced degrees should consider commencing at the graduate level. In Canada, the value of government awards for university research trainees had remained stagnant for decades [21]. In 2024, the country announced its first increase in postgraduate researcher pay in over 20 years–a positive step forward [22]. However, this increase applies only to federally administered scholarships and fellowships, benefiting only a small portion of graduate students. Future funding adjustments should ensure broader support for all graduate students and, at a minimum, keep pace with inflation. As we have aimed to illustrate, the return on investment in training is realized by defining pathways for scientists to enter the policy workforce and make science-informed policy contributions.

In the absence of formal training, it is important for scientists and students to seek opportunities for science policy engagement during their research and/or training. Potential actions include starting locally by organizing outreach events with community partners, engaging in local governmental processes, seeking science policy networks, and advocating for public policy considerations in their university developments and/or professional societies [17, 19, 23]. Additionally, scientists and students should adopt a lens toward policy by considering the real-world implications of their work.

Finally, we note the importance of evaluation of the fellowship, which was absent from our experience. Establishing a process for feedback–such as an exit interview or survey–can provide senior leadership, funders, and program implementers valuable insights into strengths and areas for improvement, supporting informed decisions on the future of these efforts.

## Conclusion

As with [24], our experience affirms that formal science policy fellowships serve as a valuable entry point for trainees seeking opportunities outside of traditional academic roles. SR continues to work within a science-based government department, while SL works for an international research institute focused on supporting policy with scientific evidence. Additionally, this experience allowed us to contribute to translating evidence into public policies, ultimately benefiting society. While science is widely recognized as important, there are risks of it being undermined. Science policy training could help to ensure science is taken seriously.
